# Boosting Quantification of N‑Glycans by an Enhanced Isobaric Multiplex Reagents for Carbonyl-Containing Compound (SUGAR) Tagging Strategy

**DOI:** 10.1021/jasms.5c00153

**Published:** 2025-08-11

**Authors:** Jingwei Zhang, Zicong Wang, Yuan Liu, Henrik Zetterberg, Lingjun Li

**Affiliations:** † Department of Chemistry, 5228 University of Wisconsin-Madison, Madison, Wisconsin 53706, United States; ‡ School of Pharmacy, University of Wisconsin-Madison, Madison, Wisconsin 53705, United States; § Institute of Neuroscience and Physiology, Sahlgrenska Academy, University of Gothenburg, Gothenburg 43141, Sweden; ∥ Clinical Neurochemistry Laboratory, Sahlgrenska University Hospital, Mölndal 43130, Sweden; ⊥ Department of Neurodegenerative Disease, UCL Institute of Neurology, London WC1N 3BG, U.K.; # UK Dementia Research Institute at UCL, London WC1N 3BG, U.K.; 7 Hong Kong Center for Neurodegenerative Diseases, Clear Water Bay, Hong Kong 999077, China; 8 Wisconsin Alzheimer’s Disease Research Center, University of Wisconsin School of Medicine and Public Health, University of Wisconsin-Madison, Madison, Wisconsin 53792, United States; 9 Centre for Brain Research, Indian Institute of Science, Bangalore 560012, India; 10 Lachman Institute for Pharmaceutical Development, School of Pharmacy, University of Wisconsin-Madison, Madison, Wisconsin 53705, United States; 11 Wisconsin Center for NanoBioSystems, School of Pharmacy, University of Wisconsin-Madison, Madison, Wisconsin 53705, United States

## Abstract

Glycans are complex molecules composed of various monosaccharides and exhibit diverse, branched polymer structures. Extensive research has been conducted on mass spectrometry (MS)-based qualitative and quantitative glycan analysis due to their critical biological functions. However, traditional data-dependent acquisition (DDA) in MS analysis primarily selects a limited subset of abundant ions during MS1 scans for fragmentation in subsequent MS2 stages. In this study, we introduce an advanced isobaric labeling strategy that incorporates a large amount of content-relevant sample labeled with one isobaric tag channel as an additional boosting channel. This innovation enhances the efficiency of isobaric multiplex reagents for carbonyl-containing compound (SUGAR) tagging in quantitative glycomics. Notably, this approach significantly improves the characterization of low-abundance N-glycans and enables the detection of subtle quantitative differences in N-glycan profiling.

## Graphical abstract

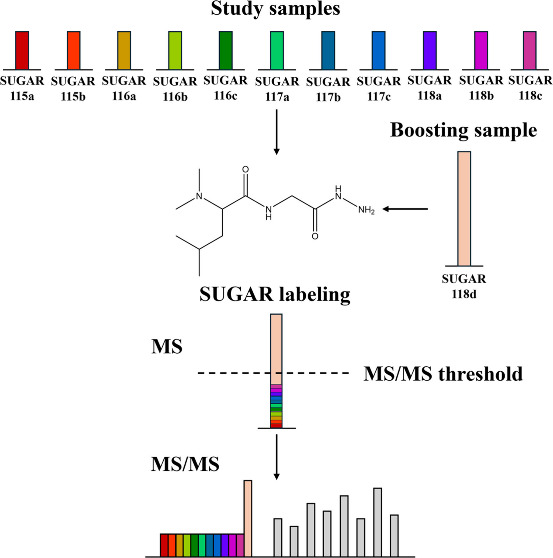

## 1 Introduction

Protein glycosylation is a widespread and functionally diverse posttranslational modification (PTM) that is essential for numerous physiological and pathological processes.^1^ Glycans contribute to key cellular functions, including cell–cell communication, cell trafficking, and protein solubility.^2^ Protein glycosylation is broadly classified into two types: N-glycosylation and O-glycosylation. Specifically, N-glycans are attached to specific amino acid sequence motifs, Asn-X-Ser/Thr, where X represents any amino acid except proline. Structurally, N-glycans share a common core of GlcNAc2Man3. Due to microheterogeneity in glycan structures, these modifications are significantly larger and more complex than other common PTMs. This complexity arises not only from variations in monosaccharide composition-mammalian glycoproteins commonly incorporate nine different monosaccharides during glycosylation but also from differences in linkages, stereochemistry, and branching patterns.^3^ Such structural changes can influence protein function and have been linked to various diseases, including cancers and neurological disorders such as Alzheimer’s disease (AD).^4–6^ Growing evidence suggests that protein glycosylation plays a crucial role in various biological processes and undergoes significant changes during disease progression.^7^ Glycomics has emerged as a promising approach for identifying diagnostic biomarkers in circulating biofluids for brain-related diseases.^8^ Targeted glycomics studies on human serum from patients with such conditions offer a valuable advantage, as sample collection involves minimally invasive procedures, making it a practical and accessible method for disease assessment.^9,10^ Multiple large-scale studies have profiled the serum N-glycome in extensive human cohorts, highlighting its potential as a rich source of biomarkers.^11,12^

Mass spectrometry (MS) is widely utilized in glycomics due to its high sensitivity and capacity to generate structurally rich data.^13^ However, native glycans exhibit poor ionization efficiency and are significantly influenced by matrix effects and competitive ionization, posing challenges for MS analysis.^14^ These limitations can be effectively mitigated through chemical labeling with suitable tags or by integrating MS with separation techniques such as high-performance liquid chromatography (HPLC) or capillary electrophoresis (CE).^15,16^ Notably, the characterization of low-abundance glycans remains particularly challenging due to the high dynamic range of glycans, making their detection and analysis more difficult.^17^

The low MS signal intensity of low-abundance glycans often results in limited glycome coverage in LC-MS/MS analysis,^1^ as poor-quality MS/MS spectra fail to generate confident glycan identifications. Isobaric labeling offers a solution by enhancing MS detection sensitivity in data-dependent acquisition (DDA) mode.^18^ In this approach, the signal intensities of precursor ions in the full scan are combined across all labeling channels for the same glycan species, improving detection. The presence of abundant glycan fragments and increased MS/MS signal intensities further facilitates glycan identification while enabling multiplex quantification. Recent studies have leveraged isobaric labeling by introducing a “boosting” (or “carrier”) channel,^19–21^ where a large amount of content-relevant sample is labeled with one isobaric tag channel and combined with smaller-amount of samples labeled with the remaining multiplex tag channels. This strategy significantly amplifies the combined signal intensity of a given glycan, enhancing the detection of low-abundance glycans and mitigating sample loss effects during preparation, particularly for low-volume samples. The boosting approach has demonstrated great potential in increasing proteome coverage and enabling the quantification of low-abundance proteins and peptides, making it especially useful for PTM analysis in size-limited samples.^22,23^ However, this strategy has yet to be applied to glycan analysis. This study specifically focuses on the glycosylation of human proteins. Isobaric labeling boosts MS1 signal intensity by aggregating signals from all channels labeling the same glycan species, due to their identical *m*/*z* values. This increases precursor ion intensity and enhances B/Y fragment signals in MS/MS, improving glycan identification, isomer differentiation, and glycome coverage (Figure S1).

Our group previously developed isobaric multiplex reagents for carbonyl-containing compound (SUGAR) tags for quantitative glycomics analysis.^24,25^ To enable conjugation with the reducing ends of glycans, the SUGAR tag structure incorporates a hydrazide as the reactive group and glycine as a balancer. Compared to commercially available tags such as aminoxyTMT, iART, and QUANTITY, SUGAR is significantly more cost-effective and can be synthesized in-house with high yields.^26^ Originally designed as a 4-plex set, SUGAR has since been expanded to 12-plex by incorporating the neutron-encoding (NeuCode) strategy.^25,27^ This approach leverages the subtle mass differences between isotopes, which arise from slight variations in nuclear binding energy. In the 12-plex SUGAR system, reporter ions are distributed across four regions spanning *m*/*z* 115 to *m*/*z* 118. SUGAR tags are particularly well-suited for developing a boosting strategy for N-glycans, as they have been successfully applied to glycomic quantification in both cell lines and patient serum samples in our previous studies.^28^ Notably, the NeuCode strategy reduces the risk of isotopic impurity “leakage”,^29^ thereby improving the accuracy and reliability of quantitative glycomics analysis.

In this study, we developed a 12-plex SUGAR tag-based boosting strategy (Boost-SUGAR) for comprehensive quantitative N-glycomic analysis of size-limited samples (Figure 1). This strategy was carefully optimized by fine-tuning the boosting-to-study channel (B/S) ratios and refining instrumental parameters, including automatic gain control (AGC) and ion injection time, to enhance data acquisition efficiency. By integrating HILIC enrichment, we successfully achieved large-scale global N-glycome mapping from small amounts of bovine thyroglobulin (BTG) and human serum. Ion mobility spectrometry coupled with mass spectrometry (IM–MS) has gained significant attention as a powerful analytical technique for enhancing glycan characterization, particularly due to its ability to separate isomeric glycans.^30,31^ Here, we evaluated the impact of high-field asymmetric waveform ion mobility spectrometry (FAIMS) by examining how different compensation voltage (CV) settings influence N-glycan identification. To further validate the feasibility of Boost-SUGAR for analyzing size-limited clinical samples, we applied this strategy to quantify N-glycome alterations in serum from AD patients compared to non-AD donors. Overall, the Boost-SUGAR strategy not only expanded glycome coverage but also enabled accurate and robust quantification, highlighting its potential for future applications in quantitative glycomics involving samples of limited availability.

**Figure 1:**
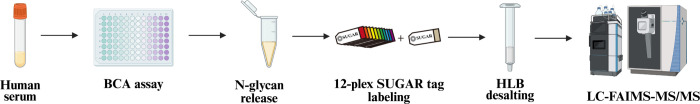
Workflow for the relative quantification of SUGAR-labeled N-glycans illustrating the stepwise experimental method. In the 12-plex SUGAR tag labeling, each channel is represented by a different color, with the boosting channel specifically highlighted in khaki.

## 2 Materials and Methods

### 2.1 Materials

Acetic acid (AA), acetonitrile (ACN), dimethyl sulfoxide (DMSO), formic acid (FA), methanol (MeOH), and water were purchased from Fisher Scientific (Pittsburgh, PA). Glycine, glycine-2,2-*d*_2_, leucine, 1-^13^C, ^15^N-leucine, 1,2-^13^C_2_-leucine, 2-^13^C, ^15^N-leucine, formaldehyde, formaldehyde-d_2_ solution, sodium cyanoborohydride, sodium cyanoborodeuteride, triethylammonium bicarbonate buffer (TEAB, 1.0 M), tris­(2-carboxy-ethyl)­phosphine hydrochloride (TCEP) and human serum were purchased from Sigma-Aldrich (St. Louis, MO). PNGase F was purchased from Bulldog Bio (Portsmouth, NH). Bovine thyroglobulin (BTG) was purchased from Thermo Fisher Scientific (Rockford, IL). Oasis HLB 1 cm^3^ cartridges were purchased from Waters Corporation (Milford, MA). Microcon-30 kDa centrifugal filters (30K MWCO) were purchased from Merck Millipore Ltd. (Darmstadt, Germany). PolyGLYCOPLEX A beads (3 μm) were purchased from PolyLC Inc. (Columbia, MD). Fused silica capillary tubing (i.d., 75 μm, o.d., 375 μm) was purchased from Polymicro Technologies (Phoenix, AZ). All reagents were used without additional purification.

### 2.2 N-Glycan Release by Filter-Aided N-Glycan Separation (FANGS)

The release of N-glycans using PNGase F was adapted from the FANGS protocol with minor modifications. Briefly, BTG and human serum proteins were dissolved at 1 mg/mL in 0.5 M TEAB buffer containing 25 mM TCEP and then heat-denatured. The proteins were subsequently transferred onto 30 kDa MWCO filters, followed by three rounds of buffer exchange with 0.5 M TEAB. PNGase F was then added at a 1:50 enzyme-to-protein ratio, and the mixture was incubated at 37 °C overnight for glycan release. A similar workflow was applied to AD serum samples. After enzymatic digestion, the filters were washed three times with 200 μL of 0.5 M TEAB buffer, and the collected fractions were dried in vacuo with SpeedVac and then treated with 1% AA at room temperature for 4 h to convert glycosylamines into glycans with free reducing ends. Finally, the samples were dried in vacuo before proceeding with labeling.

### 2.3 N-Glycans Labeled by 12-plex SUGAR Tags

12-plex SUGAR tag was synthesized in-house accordingly with previous publication.^24,25^ The structure and synthesis route of 12-plex SUGAR tag were provided in Figure S2 and S3. Released N-glycans from 200ug glycoprotein were mixed with 1 mg of the SUGAR tag in 100 μL of MeOH containing 2% FA, incubated for 15 min, and then dried *in vacuo*. This step was repeated with 100 μL of MeOH containing 1% FA followed by another round of vacuum drying. Next, 100 μL of reductive buffer containing 1 M NaBH_3_CN in DMSO:AA (7:3 v/v) was added, and the reaction was carried out at 70 °C for 2 h. To remove excess SUGAR tags and chemical reagents, an Oasis HLB 1 cm^3^ cartridge was used. The cartridge was preconditioned sequentially with 1 mL of 95% ACN, 1 mL of water, and another 1 mL of 95% ACN. The reaction mixture was then loaded onto the prefilled cartridge containing 1 mL of 95% ACN, followed by three washes with 1 mL of 95% ACN. The SUGAR tag-labeled N-glycans were eluted using 1 mL of 50% ACN and 1 mL of water, and the combined eluates were dried *in vacuo*. Finally, the dried samples were reconstituted in 75% ACN and immediately analyzed by LC-MS/MS.

### 2.4 LC–MS/MS Analysis

A self-fabricated nanoflow hydrophilic interaction chromatography (HILIC) column (15 cm, 75 μm i.d., 3 μm PolyGlycoPlex A HILIC beads) was used for glycan separation. Nano LC-MS/MS analysis was performed using a Vanquish Neo UHPLC system (Thermo Scientific, Bremen, Germany) coupled to an Orbitrap Exploris 480 mass spectrometer (Thermo Scientific, Bremen, Germany) with a high-field asymmetric waveform ion mobility spectrometer (FAIMS) Pro DUO interface (Thermo Scientific, Bremen, Germany). Mobile phase A was ACN with 0.1% FA, and mobile phase B was water with 0.1% FA. The flow rate was set at 0.35 μL/min, with an injection volume of 2 μL. The gradient conditions are detailed in Table S1. For MS data acquisition, samples were ionized in positive ion mode with a spray voltage of 2200 V. The S-lens radio frequency (RF) level was set to 55, and the capillary temperature was maintained at 280 °C. Full MS scans were performed over an *m*/*z* range of 500–2000 with a resolving power of 60k (at *m*/*z* 200). The maximum injection time was set to 100 ms, with an automatic gain control (AGC) target of 5 × 10^5^ and 1 microscan per full MS scan. A Top 20 data-dependent acquisition (DDA) analysis was performed at a resolving power of 60k (at *m*/*z* 200) using higher-energy collisional dissociation (HCD) with a normalized collision energy of 30 ± 10. A dynamic exclusion window of 15 s was applied, with a ±10 ppm precursor tolerance. Standard resolution mode was applied in FAIMS with a total carrier gas flow of 4.6 L/min.

### 2.5 N-Glycan Data Analysis

Raw mass spectrometry data were analyzed against an in-house glycan database containing potential combinations of N-glycan structural units, including hexose (H), N-acetyl hexosamine (N), fucose (F), and N-acetylneuraminic acid (S, NeuAc). Identification of 12-plex SUGAR-labeled N-glycans was first performed by accurate mass matching of precursor ions in the full MS spectra within a mass tolerance of ± 10 ppm. Subsequently, glycan structures were validated manually by inspecting glycan fragment ions (diagnostic B and Y ions) in the MS/MS spectra using GlycoWorkbench software.^32^ Reporter ion intensities corresponding to each validated SUGAR-labeled glycan were extracted with a ± 1 mDa mass tolerance. Quantitative data were processed and visualized using Microsoft Excel and GraphPad Prism 9 software.

## 3 Results and Discussion

### 3.1 Assessment of the Performance of the Boost-SUGAR Strategy

In our Boost-SUGAR strategy, a dedicated boosting channel was constructed by labeling a substantially larger amount of glycan sample (e.g., 10–20-fold greater) relative to the individual study channels, which contained small, limited amounts of sample. After combining, isobaric labeled glycans from all channels coalesced into a single precursor ion at the MS1 level. The boosted precursor intensities significantly improved the efficiency and quality of subsequent MS2 fragmentation, thereby enhancing both detection sensitivity and quantification accuracy, especially for low-abundance glycans.

To validate the Boost-SUGAR strategy and systematically evaluate the impact of varying the boosting-to-study channel (B/S) ratios, a series of experiments were conducted using human serum standard. In these experiments, the first three channels of the 12-plex SUGAR tag (115a, 115b, and 116a) were designated as study channels, each loaded with N-glycans released from 320 ng of serum protein. The last channel, SUGAR 118d, was designated as the boosting channel, and used to create B/S ratios of 5×, 10×, and 20× relative to each study channel (Figure 2A). Additionally, a control group consisting only of the three study channels without a boosting channel, was prepared in parallel. After labeling, all sample groups were pooled accordingly and subjected to LC-MS/MS analysis. SUGAR 118d was chosen as the boosting channel due to its minimal isotopic interference with adjacent reporter channels. The −1 isotopic peak of the 118d reporter ion (*m*/*z* 117.1465) is separated by 3 mDa from the nearest reporter ion, 117c (*m*/*z* 117.1436), allowing clear differentiation by high-resolution Orbitrap LC-MS/MS. As shown in Figure 2B, the reporter ion intensity distribution at a 20× B/S ratio confirms consistent signal levels across the study channels, with no abnormal signal detected in unused channels. These results demonstrate that channel 118d effectively functions as a boosting channel without causing isotopic interference, thereby supporting accurate quantification and preserving the full multiplexing capacity of the SUGAR tag system.

**Figure 2:**
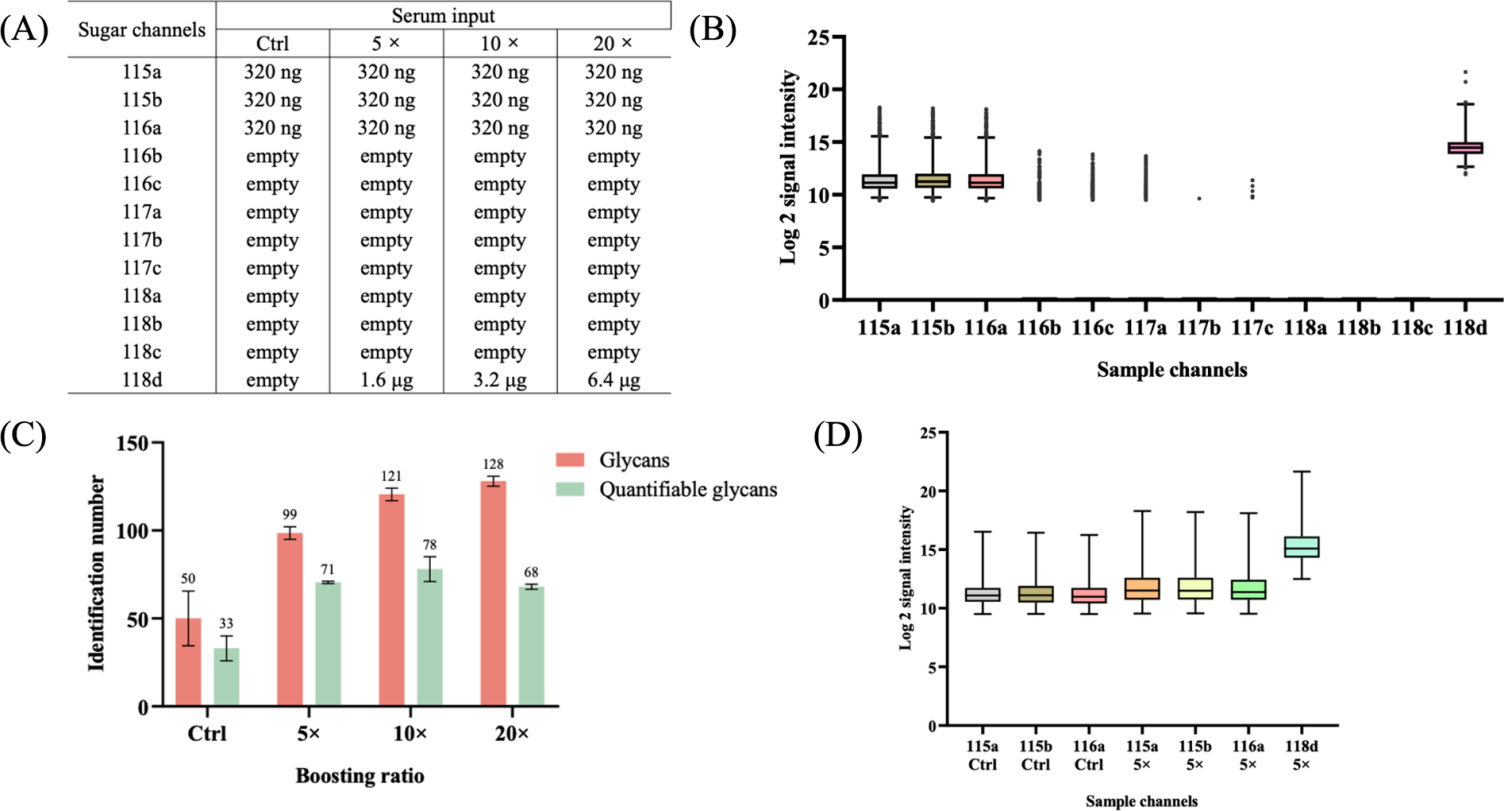
(A) SUGAR channel assignment in ctrl, 5×, 10×, and 20× experiments. (B) Distribution of reporter ion signal intensities across 12 channels. (C) Quantifiable N-glycans with different boosting ratios. (D) Reporter ion signal intensities at ctrl and 5× boosting ratio. The center line within each box denotes the median signal intensity. The box boundaries represent the interquartile range (IQR), spanning from the 25th percentile (Q1) to the 75th percentile (Q3). Whiskers extend to the minimum and maximum values within 1.5 times the IQR from the lower and upper quartiles, respectively.

The impact of incorporating a boosting channel is demonstrated in Figure 2C, which shows that the number of quantifiable glycans—defined as glycans displaying detectable reporter ion intensities across all study channels—increased 2-fold at the 5× B/S ratio compared to the control group. As expected, the total number of identified glycans increased from 99 to 128 with an increase in B/S ratios to 20x. However, the number of quantifiable glycans decreased at 20x B/S ratios. This is likely due to the fixed ion capacity of the Orbitrap during each scan, where ions from the boosting channel can dominate, reducing the relative abundance of ions from the study channels. These findings suggest that a 10× boosting ratio offers the optimal balance between glycan identification and quantification accuracy. Additionally, reporter ion intensities were notably elevated at a 5 × boosting ratio compared to the control group (Figure 2D), further underscoring the effectiveness of the boosting strategy in enhancing glycan detection sensitivity. Collectively, these findings demonstrate that the Boost-SUGAR strategy significantly improves glycomic coverage.

### 3.2 Optimization of Instrument Parameters

During MS/MS analysis, the number of precursor ions that enter the Orbitrap analyzer is governed by two key parameters: the automatic gain control (AGC) setting and the maximum injection time. These settings are crucial for balancing detection sensitivity and the MS2 scan rate. In global proteomics, AGC is typically set between 5E4 and 1E6 to maximize coverage.^20^ However, such settings may not be optimal for glycan analysis, particularly when boosting samples dominate the ion population, potentially suppressing signals from the study channels.^3,33^

To determine the optimal AGC setting for glycomics analysis with Boost-SUGAR, we systematically compared four AGC settings (1E4, 1E5, 5E5, and 1E6) using human serum-derived SUGAR-labeled glycans at a fixed boosting ratio of 10× and a consistent maximum injection time of 150 ms (Figure 3A). As expected, higher AGC values permitted greater ion accumulation, thereby enhancing the sensitivity and quality of MS/MS spectra but resulting in slightly slower MS2 acquisition rates. Among these settings, an AGC of 1E6 yielded the highest number of both identified and quantifiable glycans. Lower AGC settings (1E4, 1E5, and 5E5) generated similar glycan identification numbers, though the 5E5 setting provided notably higher number of quantifiable glycans than 1E4 and 1E5, likely due to improved sampling of study channel ions. The lowest tested setting (AGC = 1E4) significantly reduced glycome coverage, emphasizing the necessity for adequate ion accumulation to produce high-quality spectra. Furthermore, increased AGC settings enhanced reporter ion intensities, thus benefiting quantification accuracy by providing stronger signals from the study channels (Figure 3B).

**Figure 3:**
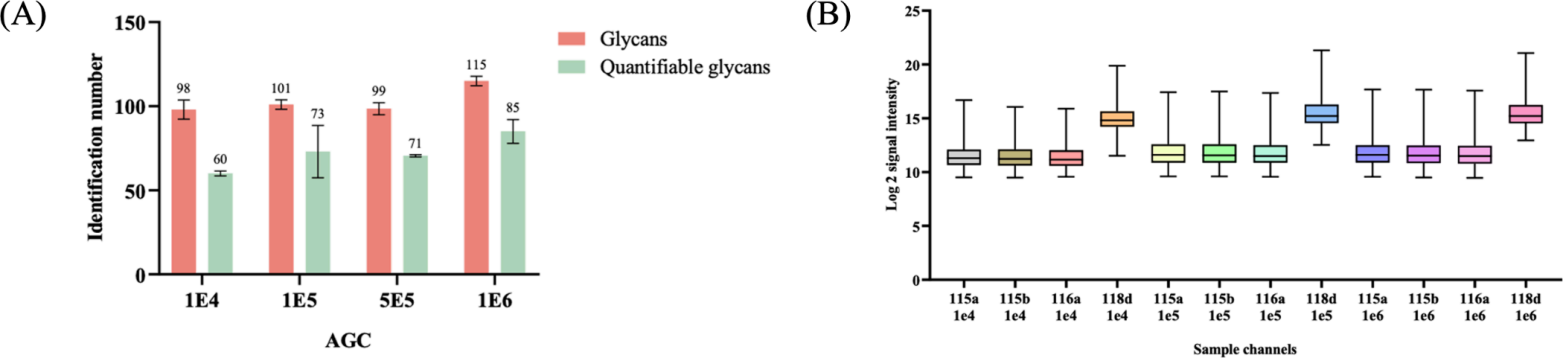
Comparison of different AGC settings. (A) Identification number of total N-glycans and quantifiable N-glycans with different AGCs. (B) Distribution of reporter ion signal intensities. The center line within each box denotes the median signal intensity. The box boundaries represent the interquartile range (IQR), spanning from the 25th percentile (Q1) to the 75th percentile (Q3). Whiskers extend to the minimum and maximum values within 1.5 times the IQR from the lower and upper quartiles, respectively.

In parallel, the maximum injection time was also optimized, as it directly influences the number of ions accumulated for MS2 fragmentation. Two injection times—50 and 250 ms—were evaluated at a fixed AGC target of 1E6 and a boosting ratio of 10×. Increasing the injection time to 250 ms improved the number of quantified glycans (Figure S4), reflecting enhanced analytical sensitivity. Considering these results, we selected an AGC target of 1E6 combined with a 250 ms injection time as the optimal settings for subsequent glycomic analyses.

Notably, the Boost-SUGAR strategy significantly facilitated the structural differentiation of glycan isomers. Several isomeric glycans were identified based on identical precursor masses but distinct retention times and structural ions. Notably, the enhanced structural ion intensities provided by the Boost-SUGAR approach increased the detection of characteristic B/Y fragment ions (Figure 4), which are instrumental in elucidating subtle structural variations among glycan isomers. This improved MS/MS spectral quality directly translated into clearer differentiation and identification of structural glycan isomers, further underscoring the advantage of employing the Boost-SUGAR strategy in detailed glycan structure analysis.

**Figure 4:**
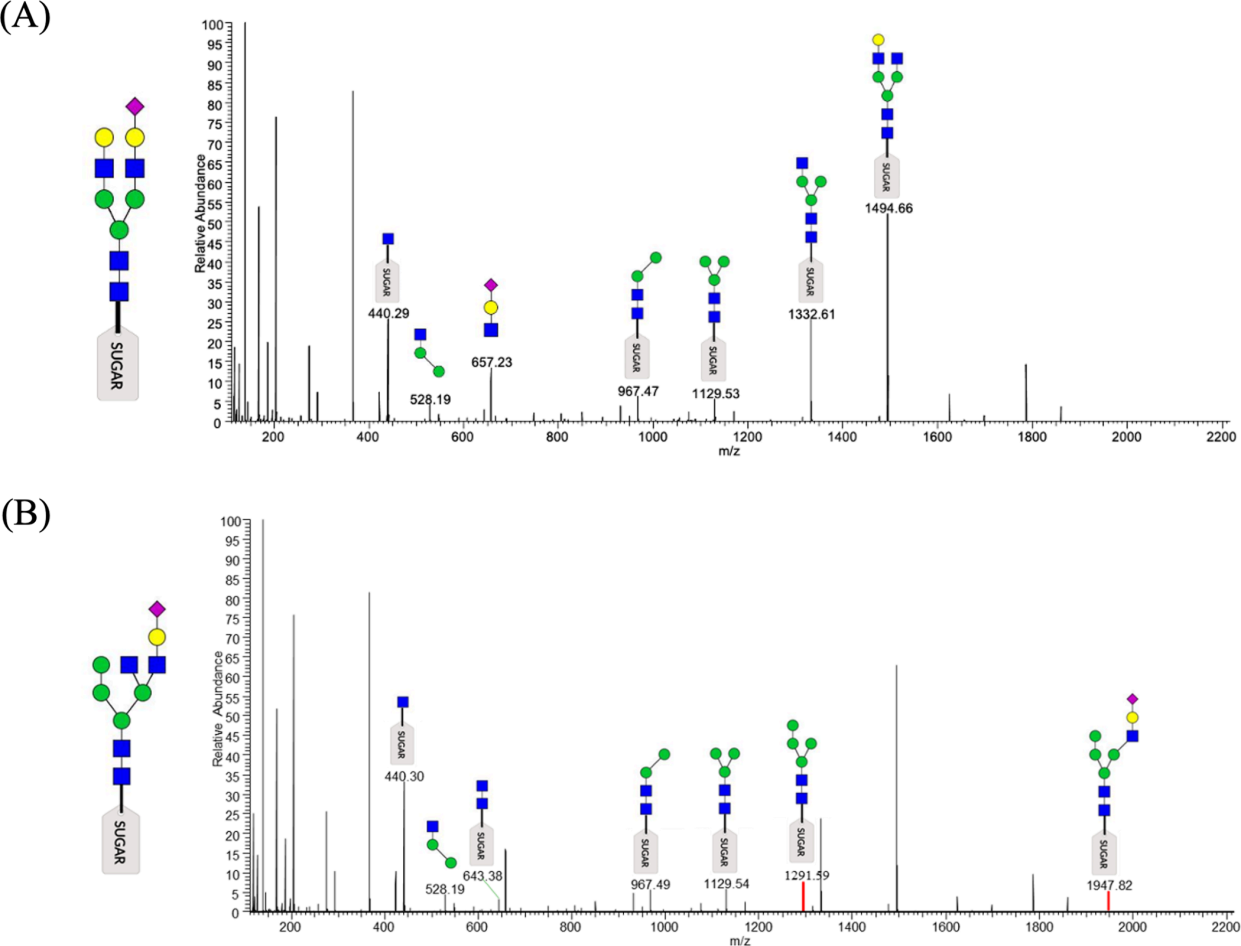
(A, B) Representative MS/MS spectra of the two isomers of the same glycan composition (N4H5S1).

### 3.3 Using FAIMS to Improve the Glycome Coverage

In addition to optimizing instrumental parameters, we investigated the use of FAIMS to further enhance glycome coverage. Ion mobility spectrometry (IMS) is a powerful gas-phase separation technique that differentiates analytes based on their charge states and collisional cross sections (CCS) values.^34,35^ Among various IMS techniques, FAIMS has been widely adopted in proteomic analyses due to its ability to increase analytical depth.^36,37^ However, the benefits of FAIMS for glycan analysis have not yet been systematically explored. This study represents the first investigation of glycome coverage using FAIMS. To evaluate the effect of FAIMS CV settings on N-glycan identification, we analyzed SUGAR-labeled N-glycans enriched from human serum using CVs ranging from −25 V to −65 V in 10 V increments. As shown in Figure 5, due to the relatively large collisional cross-section of N-glycans compared to unmodified peptides, lower FAIMS CV settings appeared to be more favorable for glycan detection. Among single CV settings, −35 V, −45 V, and −55 V yielded the highest number of identified N-glycans (Figure 5A). We also tested various combinations of CVs, and the greatest glycan coverage was achieved using a combination of −25 V, −35 V, and −45 V (Figure 5B). As a result, this triple CV setting was selected for all subsequent experiments. We also compared glycome coverage with and without the use of FAIMS, and observed improved coverage with FAIMS, as illustrated in Figure S5.

**Figure 5:**
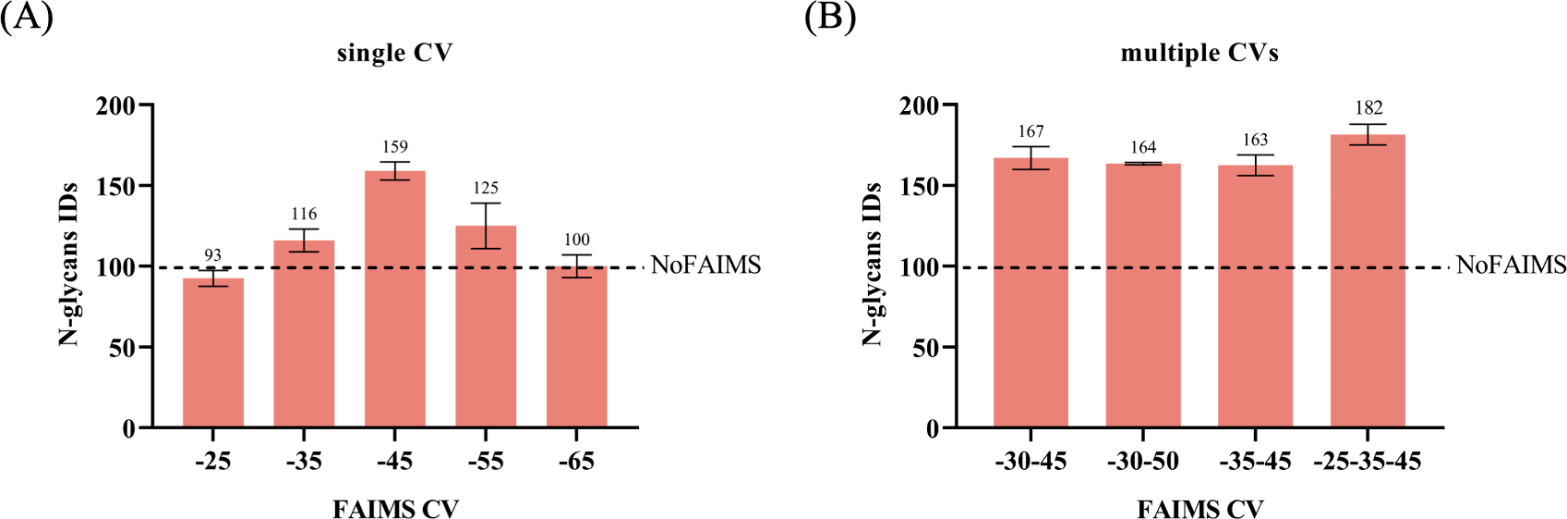
Evaluation and optimization of FAIMS CV settings for identifying N-glycans derived from human serum. (A) N-Glycan identification number with single CVs. (B) N-Glycan identification number with double CVs and triple CVs.

### 3.4 Quantitative Glycomic Analysis of Human Serum Samples in AD

Finally, we applied the Boost-SUGAR strategy to investigate glycosylation changes in human serum associated with AD. Although human serum is a rich source of biochemical information, its high dynamic protein range poses significant challenges for glycomics, as abundant proteins can obscure the detection of low-abundance glycans.^10^ In proteomics, high-abundance protein depletion is commonly used to improve detection sensitivity in complex fluids such as plasma, serum, and cerebrospinal fluid (CSF).^38,39^ However, this approach often leads to codepletion of proteins bound to albumin or IgG, which may result in the loss of potential biomarkers.^40,41^ Such an approach requires a large amount of starting material to obtain sufficient sample for analysis.

To address these limitations, we utilized the Boost-SUGAR strategy to enhance the detection of low-abundance N-glycans in AD serum samples. After quantifying protein concentrations with a BCA assay, N-glycans were enzymatically released from 100 μg of serum protein from AD patients and non-AD donors. The released glycans were then labeled using 12-plex SUGAR tags, following the established protocol, with channel 118d designated as the boosting channel. For further quantification, N-glycans lacking appreciable signal intensities were excluded, resulting in 124 confidently quantified N-glycans. To illustrate differences in glycan expression, box plots were generated, highlighting four N-glycans with the most significant upregulation (Figure 6). We observed notable overexpression of several glycans in AD samples, including H5N5F2 (Figure 6A), H6N5F3S1 (Figure 6B), H6N5F1S3 (Figure 6C), and H6N5F4S2 (Figure 6D).

**Figure 6:**
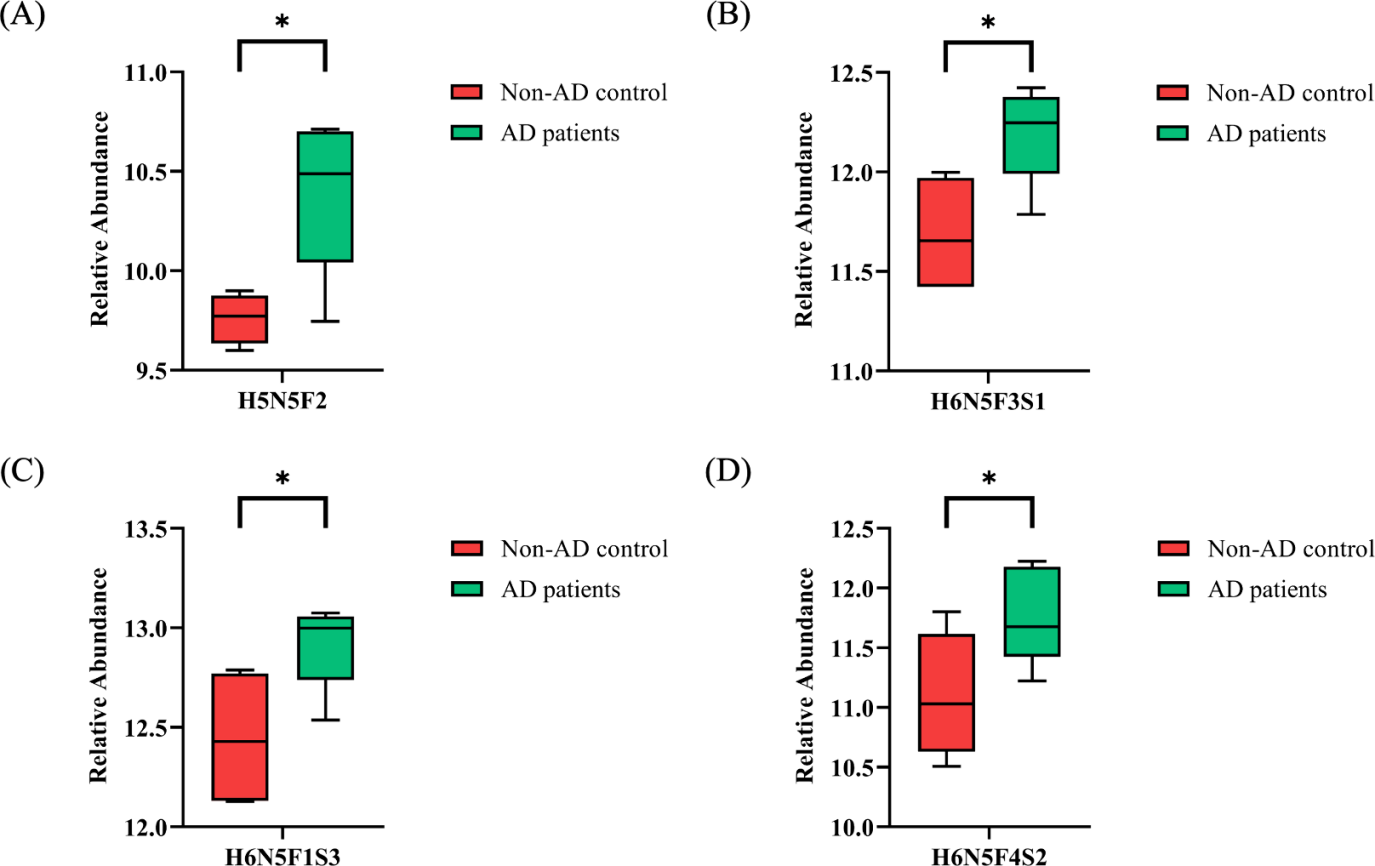
Box plots depict the comparison between relative abundances of N-glycan structures H5N5F2 (A), H6N5F3S1 (B), H6N5F1S3 (C), and H6N5F4S2 (D) in serum samples from five non-AD control individuals and five AD patients (please see Supporting Information for details). Significant difference was determined by a two-tailed *t* test (*p < 0.05, and **p < 0.01).

These four glycans exemplify the elevated fucosylated and sialylated N-glycans in AD serum, aligning with prior glycomics studies. For example, Lebrilla and co-workers demonstrated that AD patients exhibit global increases in both fucosylation and sialylation on key serum glycoproteins—including immunoglobulins and complement factors—linking aberrant glycosyltransferase activity to Aβ protein aggregation, tau phosphorylation, and chronic neuroinflammation.^42^ Likewise, Zhou et al. identified hyper-branched di- and trisialylated N-glycans as early blood biomarkers that predict impending cognitive decline, consistent with our observed upregulation of H6N5F1S3 in AD serum.^11^ Taken together, the glycan changes captured by our Boost-SUGAR workflow provide preliminary insights into the potential utility of N-glycan profiling in differentiating serum samples from individuals diagnosed with AD versus non-AD controls. However, due to the limitations of the current study—including the absence of detailed clinical diagnostic criteria, lack of information on disease staging, potential confounding health conditions (such as cardiovascular diseases and diabetes), uncontrolled factors associated with serum sample processing, and unknown analytical randomization—these observations must be interpreted with caution. Determining whether these glycan alterations directly reflect mechanisms of AD or represent secondary epiphenomena or experimental bias is beyond the scope of the present exploratory research. Future studies with well-characterized clinical cohorts, standardized sample preparation protocols, rigorous control of confounding factors, and randomized analytical approaches are essential to validate the disease specificity and mechanistic significance of these preliminary findings.

## 4 Conclusions

In this study, we developed a Boost-SUGAR strategy that enhances MS signal intensity through isobaric labeling, enabling highly sensitive characterization of N-glycans from human serum. Key parameters—including the N-glycan release protocol, labeling conditions, boosting and study channel ratio, and MS acquisition settings—were systematically optimized to improve quantification performance. The accuracy and sensitivity of the method were first validated using BTG and human serum, and then further demonstrated in more complex biological samples. Compared to conventional DDA approaches, our strategy provided unprecedented depth in N-glycan profiling, along with informative MS2 fragments that enabled differentiation of glycan isomers. Its successful application to small-volume human serum samples from AD patients and healthy donors highlights its potential for broader use in profiling N-glycans from other biological fluids, such as CSF. The preliminary results obtained from the small set of human serum samples highlight the technical feasibility of the Boost-SUGAR strategy in detecting N-glycan differences. However, comprehensive clinical validation—including rigorous characterization of clinical samples, control of sample processing, and addressing potential confounders—is essential before these findings can be reliably associated with AD progression or utilized for biomarker discovery.

## Supplementary Material



## Notes

Supplemental Figures: Boost-SUGAR quantitative strategy (Figure S1); structure of the 12-plex SUGAR isobaric labeling system (Figure S2); synthetic route for the SUGAR tag (Figure S3); comparison of ion injection time settings on N-glycan coverage (Figure S4); Venn diagram illustrating overlap of identified glycans with and without FAIMS (Figure S5). Supplemental Tables: LC gradient used for all LC-MS/MS analyses (Table S1). Information on serum samples collected from AD and non-AD subjects (Table S2) (PDF)
