# Supplementary material for: Boosting Quantification of N‑Glycans by an Enhanced Isobaric Multiplex Reagents for Carbonyl-Containing Compound (SUGAR) Tagging Strategy
Source: J Am Soc Mass Spectrom. 2025 Aug 11;36(9):1912–20. doi: 10.1021/jasms.5c00153 (PMC12450061; doi:10.1021/jasms.5c00153)
Supplement: Supplementary file 1 [file js5c00153_si_001.pdf]

Supporting Information

Boosting Quantification of N-glycans by Enhanced Isobaric Multiplex Reagents for Carbonyl-Containing Compound (SUGAR) Tagging Strategy

Jingwei Zhang[1,12], Zicong Wang[2,12], Yuan Liu[2], Henrik Zetterberg[3,4,5,6,7,8,9], Lingjun Li[1,2,10,11*]

[1] Department of Chemistry, University of Wisconsin-Madison, Madison, WI, 53706, USA

[2] School of Pharmacy, University of Wisconsin-Madison, Madison, WI, 53705, USA

[3] Institute of Neuroscience and Physiology, Sahlgrenska Academy, University of Gothenburg, Gothenburg 43141, Sweden

[4] Clinical Neurochemistry Laboratory, Sahlgrenska University Hospital, Mölndal 43130, Sweden

[5] Department of Neurodegenerative Disease, UCL Institute of Neurology, London WC1N 3BG, UK

[6] UK Dementia Research Institute at UCL, London WC1N 3BG, UK

[7] Hong Kong Center for Neurodegenerative Diseases, Clear Water Bay, Hong Kong 999077, China

[8] Wisconsin Alzheimer's Disease Research Center, University of Wisconsin School of Medicine and Public Health, University of Wisconsin-Madison, Madison, WI 53792, USA

[9] Centre for Brain Research, Indian Institute of Science, Bangalore 560012, India

[10] Lachman Institute for Pharmaceutical Development, School of Pharmacy, University of Wisconsin-Madison, Madison, WI 53705, USA.

[11] Wisconsin Center for NanoBioSystems, School of Pharmacy, University of Wisconsin-Madison, Madison, WI 53705, USA.

[12] These authors contributed equally: Jingwei Zhang and Zicong Wang

*Corresponding author: Lingjun Li

Email: lingjun.li@wisc.edu

Mailing address: 777 Highland Ave, Madison, WI 53705, United States

Tel: +1 608-265-8491, Fax: +1 608-262-5345

Table of Contents

Human serum sample collection

Figure S1. Boost-SUGAR quantitative strategy

Figure S2. Structures of the 12-plex SUGAR isobaric labeling system. (A) Representative HCD fragmentation of the SUGAR tag, showing CO neutral loss and reporter-ion generation. (B) Chemical structures of the 12 isotopologues (115a–118d) with sites of ^{13}C (blue), ^{15}N (red), and ^{2}H (yellow) incorporation.

Figure S3. Three-step synthetic route for the SUGAR tag

Figure S4. Comparison of ion-injection times on N-glycan coverage

Figure S5. Venn diagram comparing glycans identified with and without FAIMS

Table S1. LC gradient used for all LC-MS/MS analyses

Table S2. Information on serum samples collected from AD and non-AD subjects

AD serum sample collection

A total of 10 human serum samples (including 5 AD serum samples and 5 control serum samples) were provided by the Clinical Neurochemistry Laboratory in Gothenburg in a study approved by the regional ethics committee at the University of Gothenburg with all the patients' informed consent. All study procedures involving human subjects have been approved by the University of Wisconsin Institutional Review Board and abide by the Declaration of Helsinki principles. Clinical and cerebrospinal fluid (CSF) AD biomarker characteristics of the patients are shown in **Table S2**.

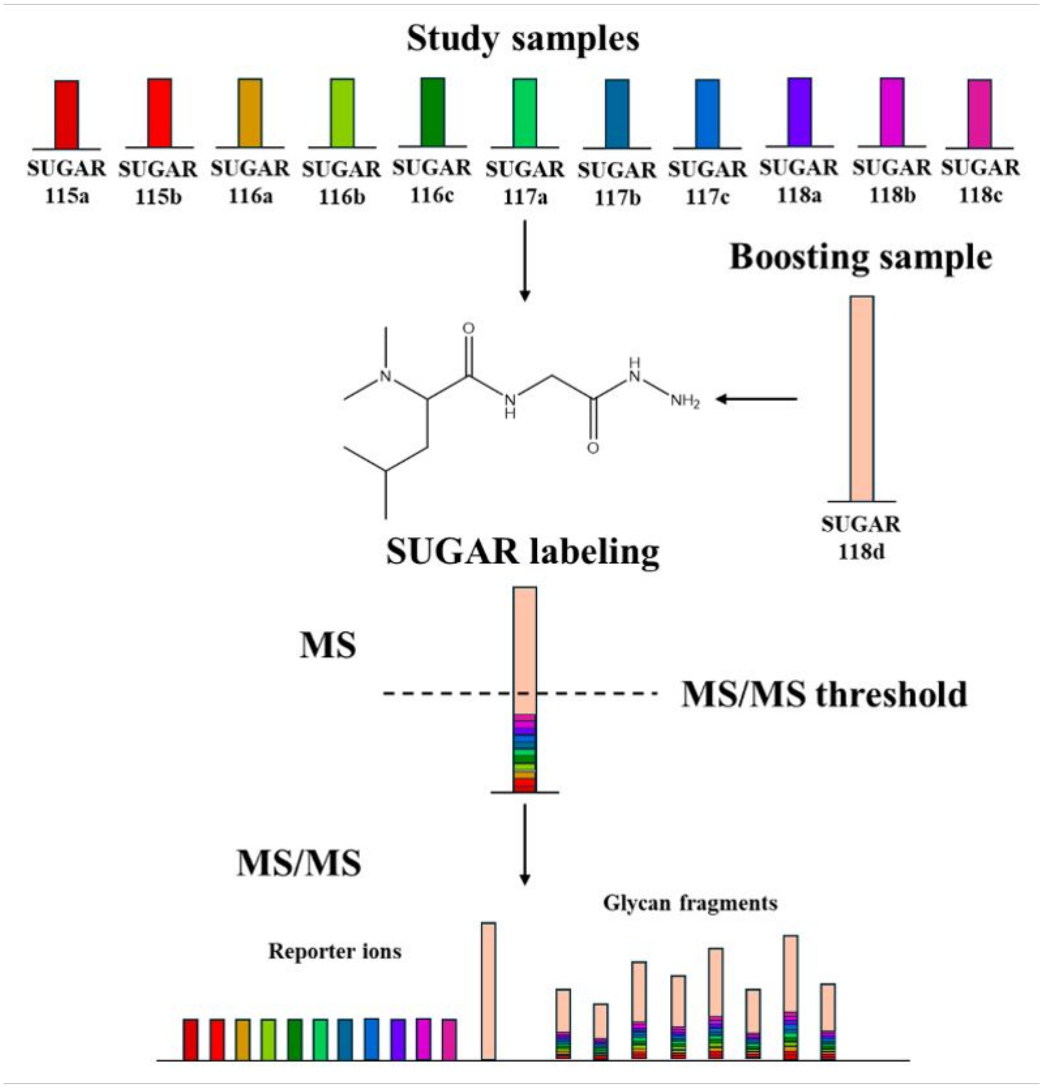

Figure S1. Boost-SUGAR quantitative strategy.

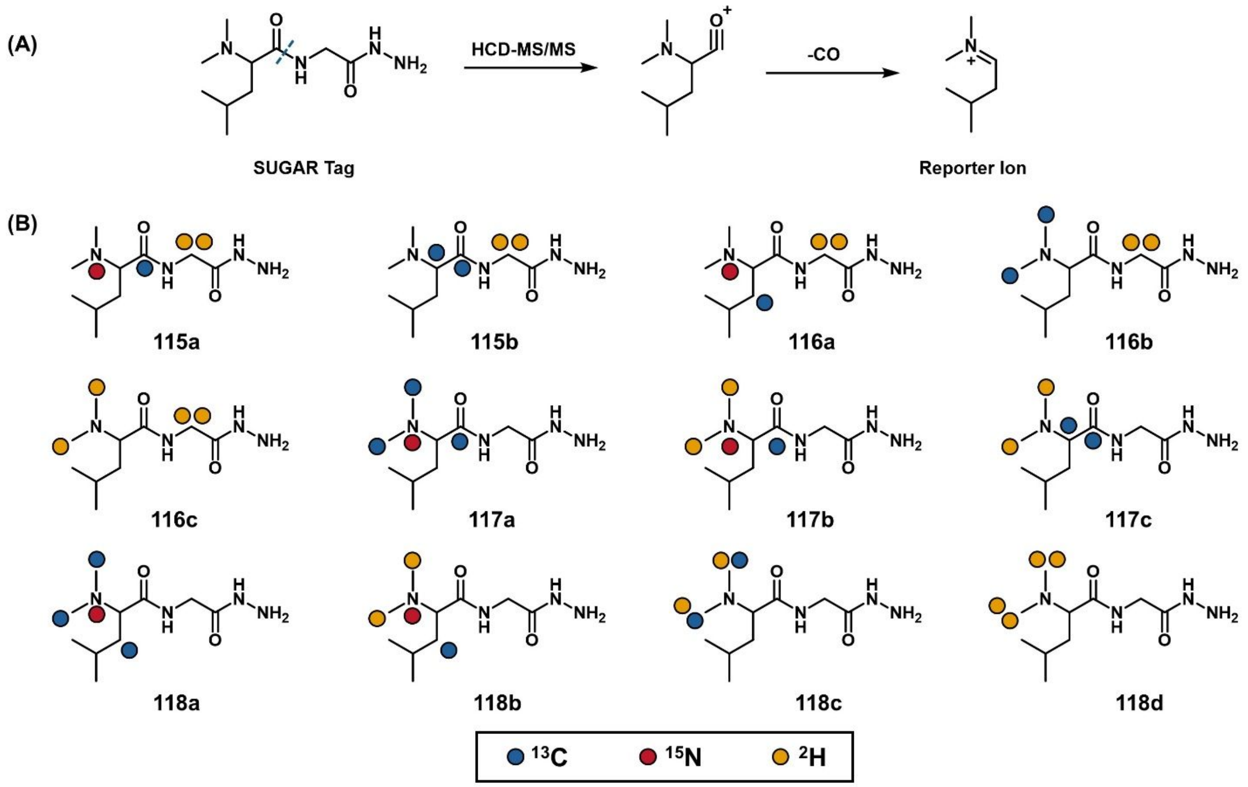

Figure S2. Structures of the 12-plex SUGAR isobaric labeling system. (A) Representative HCD fragmentation of the SUGAR tag, showing CO neutral loss and generation of the reporter ion. (B) Chemical structures of the 12 isotopologues (115a – 118d) obtained by site-specific incorporation of stable isotopes. Blue, red, and yellow circles indicate the locations of ^{13}C, ^{15}N, and ^{2}H atoms, respectively.[1]

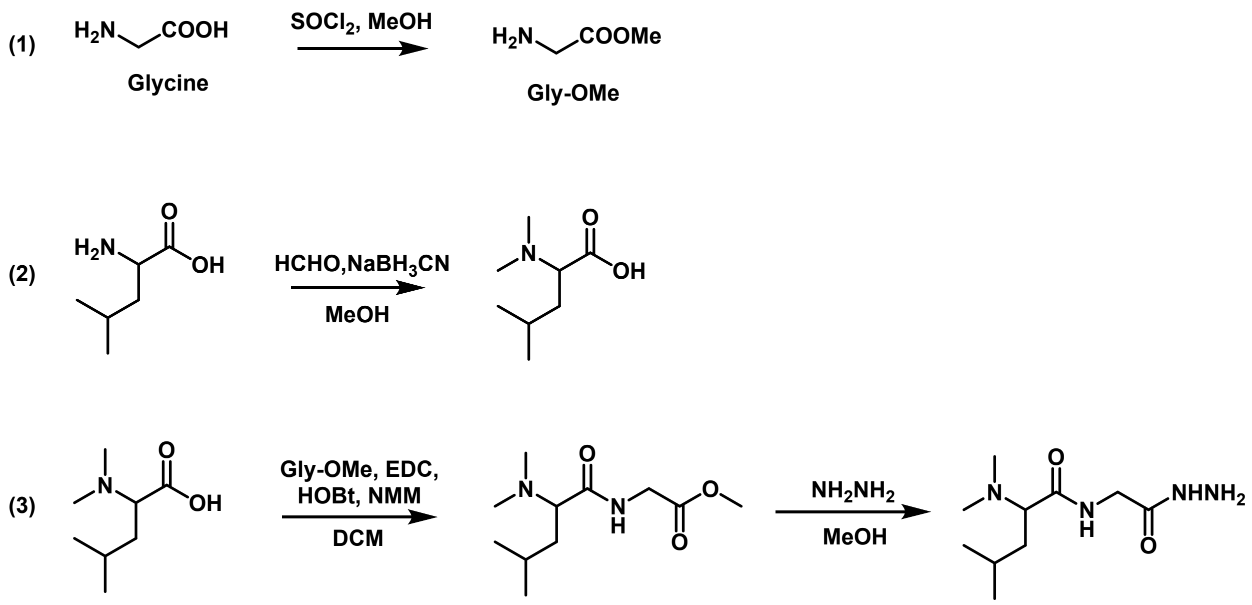

Figure S3. Synthetic route for SUGAR tag in three steps.

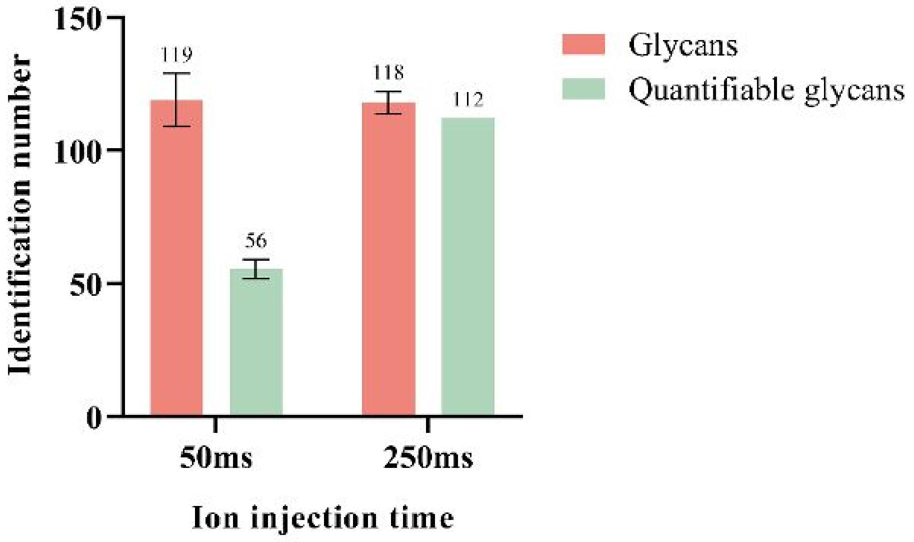

Figure S4. Comparison of different ion injection time settings on N-glycan coverage. Bar plots show the mean ± SD (n = 3) numbers of total identifications (red) and quantifiable glycans (green) obtained at 50 ms versus 250 ms injection time.

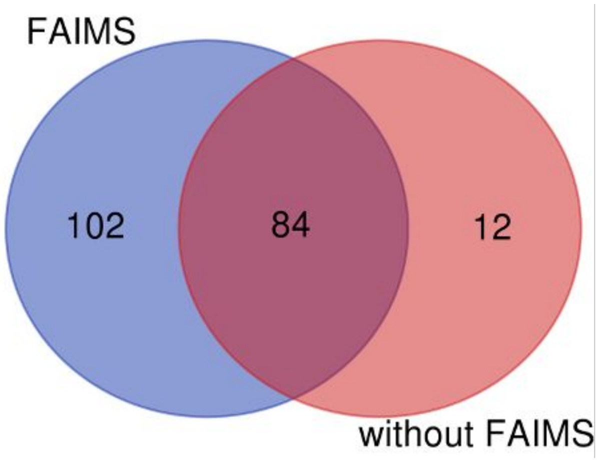

Figure S5. Venn diagram showing the overlap of identified glycans with FAIMS and without FAIMS.

Table S1. LC gradient for all LC-MS/MS analysis

TIME/MIN	% OF MOBILE PHASE B
0	25
5	25
100	70
103	85
120	85

Table S2. Information on serum samples collected from AD and non-AD subjects

Number	Age (years)	Gender	T-tau (pg/mL)	Aβ42 (pg/mL)	P-tau181 (pg/mL)	Diagnosis
1	77	male	781	274	94	AD
2	73	male	733	294	87	AD
3	75	male	777	382	100	AD
4	76	male	1160	412	142	AD
5	73	male	625	564	76	AD
6	77	male	192	624	28	non-AD
7	73	male	241	996	34	non-AD
8	75	male	300	1250	48	non-AD
9	75	male	382	830	58	non-AD
10	76	male	241	835	39	non-AD

Reference:

(1) Li, M.; Feng, Y.; Ma, M.; Kapur, A.; Patankar, M.; Li, L. High-Throughput Quantitative Glycomics Enabled by 12-Plex Isobaric Multiplex Labeling Reagents for Carbonyl-Containing Compound (SUGAR) Tags. *J. Proteome Res.* **2023**, *22* (5), 1557–1563. https://doi.org/10.1021/acs.jproteome.2c00773.
